# Association between body mass index (BMI) and [^123^I]Ioflupane (DaTSCAN) availabilities in patients with parkinsonism using single-photon emission computed tomography–computed tomography (SPECT-CT)

**DOI:** 10.1186/s41824-023-00181-6

**Published:** 2023-11-20

**Authors:** Puja Parekh, Patrick Begley, Maryam Jessop, Mark Aplin, Elena Missir, Helena McMeekin, Gosia Raczek, Nitasha Singh, Sabina Dizdarevic

**Affiliations:** 1 Clinical Imaging Science Centre, Neuroscience and Medicine, Brighton and Sussex Medical School, Brighton, England; 2 Brighton and Sussex Medical School, Brighton, England; 3 Nuclear Medicine Department, Royal Sussex County Hospital, University Hospitals Sussex NHS Foundation Trust, Brighton, England; 4 Hermes Medical Solutions, London, England

**Keywords:** DaTSCAN, BMI, Obesity, DaTQUANT, Specific binding ratios

## Abstract

**Aim:**

[^123^I]Ioflupane (DaTSCAN) has a high binding affinity to the dopamine (DA) transporter (DaT) and tenfold less affinity to serotonin (5-HT) transporter (SERT). Both neurotransmitters are considered to contribute to body weight regulation. This study assesses the association between body mass index (BMI) and DaTSCAN availability in brain.

**Method:**

Scans from 74 consecutive patients who had undergone DaTSCAN single-photon emission computed tomography–computed tomography (SPECT-CT) were used to obtain semi- and absolute quantitative data in several volumes of interest (VOIs). Relative semi-quantitative specific binding ratios (SBRs) from Chang attenuated SPECT were obtained from GE DaTQUANT. Absolute normalised concentration (NC) was calculated from attenuation/scatter corrected SPECT-CT images, using an adapted version of the EARL Ltd (European Association of Nuclear Medicine (EANM) Research 4 Life) template. Scans were subdivided into either degenerative parkinsonism (abnormal = 49), borderline (*n* = 14) or scan without evidence of dopaminergic deficit (SWEDD = 11) using visual assessment and SBR values by two nuclear medicine consultants.

**Results:**

SBRs did not correlate with BMI. However, NC values correlated negatively in the entire cohort, with the strongest correlation in the frontal (*r* = − 0.649. *p* = 0.000), occipital (*r* = − 0.555, *p* = 0.000) regions and pons (*r* = − 0.555, *p* = 0.000). In the abnormal (*n* = 49) and SWEDD group (*n* = 11), NC of the frontal region was the most correlated with BMI (*r* = − 0.570, *p* = 0.000; *r* = − 0.813, *p* = 0.002, respectively). In the borderline group (*n* = 14), the left posterior putamen displayed the strongest correlation (*r* = − 0.765, *p* = 0.001).

**Conclusion:**

Absolute NC values demonstrate a strong inverse correlation with BMI, strongest in the extrastriatal regions. Due to the predominately non-overlapping distribution of DaT and SERT, this study suggests greater involvement of SERT in obesity with possible interplay with DA transmission.

## Introduction

Obesity is the abnormal accumulation of adipose tissue and is known to be a major source of morbidity, contributing to impaired quality of life, and its related chronic complications leading to a reduced life expectancy (Abdelaal et al. 2017). Comorbidities associated with obesity include diabetes (Al-Goblan et al. 2014), cardiovascular disease (CVD) (Burke et al. 2008) and several malignancies (Pi-Sunyer 2009). The condition is due to an imbalance between energy intake and energy expenditure over long periods, resulting in excess amounts of body fat (Morton et al. 2014). Body mass index (BMI) is commonly used to measure anthropometric height/weight and utilised in clinical practice to categorise adults into groups (Nuttall 2015). Approximately, 39% and 13% of adults are defined as overweight (BMI ≥ 25 kg/m^2^) and obese (BMI ≥ 30 kg/m^2^), respectively, as reported by the World Health Organization 2021. The brain is known to play a critical role in the pathogenesis of overeating (Volkow et al. 2012) by regulating the responses to food stimuli (Volkow et al. 2012), and thus, obesity has been suggested to be included in the Diagnostic and Statistical Manual of Mental Disorders (DSM) (Devlin 2007).

Both dopamine (DA) and serotonin (5-HT) cerebral neurotransmitters have been associated with controlling food intake and are hence highly likely to contribute to the regulation of body weight (Blum et al. 2014; Lam et al. 2010). As it is currently not possible to directly measure concentrations of cerebral DA and 5-HT in vivo, neurotransmitter receptors and transporters are targeted to assess these systems using molecular neuroimaging techniques. DA transporters (DaT) and 5-HT transporters (SERT) are proteins that control the amount of intrasynaptic DA and 5-HT, and with the use of specific radiotracers, these can be visualised and quantified (Innis et al. 2007).

Previous neuroimaging studies have used single-photon emission tomography (SPECT) with [^123^I]Ioflupane (DaTSCAN) as the radioligand to investigate both DaT and SERT distributions due to its high affinity to striatal DaT (Mattsson et al. 2015) and a tenfold lower affinity to SERTs (Ziebell at al. 2010). DaTs are postulated to be densely situated in the striatum (Booij et al. 2007) and SERT within extrastriatal regions including the frontal lobe (Celada et al. 2013), thalamus (Koch et al. 2014), hypothalamus (Borgers et al. 2013), midbrain (Roselli et al. 2010; Nam et al. 2018) and pons (Koch et al. 2014). These studies suggest a non-overlapping distribution of DaTs and SERTs, allowing the measure of both with a single tracer.

Despite this, previous data have been inconsistent and contradictory when measuring the availabilities of DaT transporters in relation to obesity by SPECT and post-mortem studies (Chen et al. 2008; Wu et al. 2017; Giessen et al. 2013). However, the SPECT studies were only able to relatively quantify radiotracer binding potentials and only within the striatum and, thus, unable to absolutely measure binding in regions outside the caudate and putamen. Similarly, variable data have also been reported with SERT research (Galen et al. 2017). Due to these controversies with data regarding the function of DaT and SERTs in obesity, it is important to understand the role of these networks in individuals with high BMIs.

## Aims

This study aims to:

- Assess the association between body mass index (BMI) and DaTSCAN availability in both striatal and extrastriatal brain regions using SPECT-CT nuclear imaging scans.
- Utilise Normalised concentration (NC) values as a form of precise absolute activity quantification obtained from DaTSCAN SPECT-CT images, in contrast to specific binding ratio (SBR) obtained from SPECT only images, which represents relative semi-quantification.

## Methodology

### Ethics

This is a retrospective quality improvement study, and the Medical Research Council Health Research Authority tool (http://www.hra-decisiontools.org.uk/research/) was used to determine that the study falls under clinical audit/service evaluation. The project was approved by the Departmental Audit and Quality Improvement Group, and no ethical approval was required. Patients in this study had been referred for standard routine imaging and had not been exposed to excess radioactive material beyond the standard of care. As per routine clinical practice, all patients were verbally informed by medical professionals that their anonymised scans may be used for teaching, audit and/or research purposes.

### Participants

The study population consisted of seventy-four patients with parkinsonism symptoms who had undergone routine DaTSCAN SPECT-CT in the Nuclear Medicine Department between the 30 October 2020 and 15 October 2021 [28 females and 46 males, age range: 31–88 years, mean age: 69 years ± 12 standard deviations (SD)]. BMI category have been defined using the following cut-offs: underweight = BMI < 18.5; healthy = BMI between 18.5 and 24.9; overweight = BMI between 25 and 29.9; and obese = BMI ≥ 30. BMI range: 16.4–50.4, mean BMI: 26.69 ± 5.76 SD, 2.7% underweight BMI, 39.2% healthy BMI, 37.8% overweight BMI and 20.3% obese BMI] (Table 1). These patients had been referred to the Department of Nuclear Medicine by neurologists and old age psychiatrists to investigate for either a degenerative or non-degenerative cause of their parkinsonism. The exclusion criteria included the following: (1) unable to lie still/flat on their backs for a minimum of 45 min; (2) on medication that may interact with the visual analysis (e.g. cocaine and amphetamines); (3) pregnancy and breastfeeding; and (4) patients on selective serotonin reuptake inhibitors (SSRIs) or serotonin and norepinephrine reuptake inhibitors (SNRIs) who were not able to withdraw the treatment prior to their scans.

**Table 1:** Study population characteristics table

*n* = 74	
Sex (male/female)	46/28
Mean age ± standard deviation	69.55 ± 12.05
Mean height (metres) ± standard deviation	1.72 ± 0.09
Mean weight (kg) ± standard deviation	79.21 ± 17.99
Mean BMI (kg/m^2^) ± standard deviation	26.69 ± 5.76
BMI category (underweight/healthy/overweight/obese)	2/29/28/15
DaTSCAN subgroup (abnormal/borderline/SWEDD)	49/14/11

*n* = total number of participants included in the study

BMI = Body mass index; BMI category definitions—underweight = BMI < 18.5; healthy = BMI between 18.5 and 29.9; overweight = BMI between 25 and 29.9; and obese = BMI ≥ 30. DaTSCAN subgroups had been defined according to nuclear medicine consultant reports

### SPECT-CT protocol

Subjects were pretreated with a thyroid blocking agent approximately an hour before the intravenous administration of 185 MBq of DaTSCAN (radiolabelled cocaine analogue). Three hours after the DaTSCAN administration, SPECT-CT imaging was performed using a dual-head SPECT gamma camera model Symbia Intevo (SIEMENS Healthineers) with integrated CT (16 slices/rotation). Each patient had been immobilised using a headrest, maintaining a radial distance between 12 and 15 cm.

Raw data were acquired using low-energy, high-resolution (LEHR) collimators with the following parameters: 180° rotation step and shoot mode, 25 s per step, in a 128 × 128 image matrix with a 1.23 zoom factor.

Following the acquisition of SPECT, CT images were obtained using a helical scanning mode and the parameters: 0.6 s rotation time in the craniocaudal direction, 130 kV, 30 milliampere-seconds, pitch of 0.8 and 16 × 0.6 mm collimation. Attenuation correction maps were created by reconstructing the raw data projections twice using the following procedures (Missir et al. 2023):

- DaTQuant with the ordered subset expectation maximisation (OSEM), iterative mathematical procedure, involving 10 subsets and 2 iterations. In addition, the mathematical Chang attenuation correction (AC) (Saha et al. 2016) without scatter correction (SC) was applied.
- Hermes hybrid reconstruction was carried out with an OSEM iterative method using 5 subsets and 16 iterations with CT calculated AC, modelled SC and collimator resolution recovery. A Butterworth smoothing filter (0.7 cm^−1^/20 power) was also used to allow equivalent attenuation with less lag phase.

### Image analysis

#### BMI calculation

Weight and height measurements were obtained as per clinical protocol for all patients. The weight was divided by the square of height in metres to calculate the BMI. Participants with BMIs equivalent or greater than 30 kg/m^2^ had been classified as obese.

#### Semi-quantitative and quantitative Indexes from SPECT/SPECT-CT

##### Semi-quantitative analysis from SPECT

Patient SPECT scans were used to obtain semi-quantitative quantification values (SBRs), while SPECT-CT scans were used to obtain absolute activity quantification indices (NCs). SBRs were calculated using DaTQuant (version 2) software by GE Healthcare, to semi-quantify striata to background (occipital region) ratios of SPECT counts within the predefined volume of interests (VOI). The automated DaTSCAN template defined the bilateral caudate nuclei, anterior putamina, posterior putamina and the single occipital background region. The occipital region is considered to represent non-specific binding which is free of DaT. This was used to calculate the ratio between specific (striata) and background binding to obtain SBRs.

The formula used to calculate the SBRs for the relevant VOIs is stated below:

$${\text{SBR}} = \frac{{{\text{Specific Binding Count}}_{{\text{Relevant VOI region}}} - {\text{Specific Binding Count}}_{{\text{Background Region}}} }}{{{\text{Specific Binding Count}}_{{\text{Background Region}}} }}$$

##### Quantitative analysis from SPECT-CT

Hybrid SPECT-CT images were then used to measure absolute mean concentration within selected VOIs. The VOIs used for this study included both striatal and extrastriatal regions of the brain (frontal region, bilateral caudate nuclei, anterior putamina, posterior putamina, occipital background region, thalamus, midbrain and pons). The absolute mean concentrations were initially corrected to convert SPECT counts into the concentration of radioactivity (Bq/mL) within the VOIs. This was achieved by calculating a becquerel calibration factor (BCF) of 128.7 cps/MBq from a previous phantom study where the amount of iodine-123 and the size of the phantom container (21.3 cm diameter) were known. This BCF was then used on the subsequent reconstructed SPECT-CT.

**Fig. 1:**
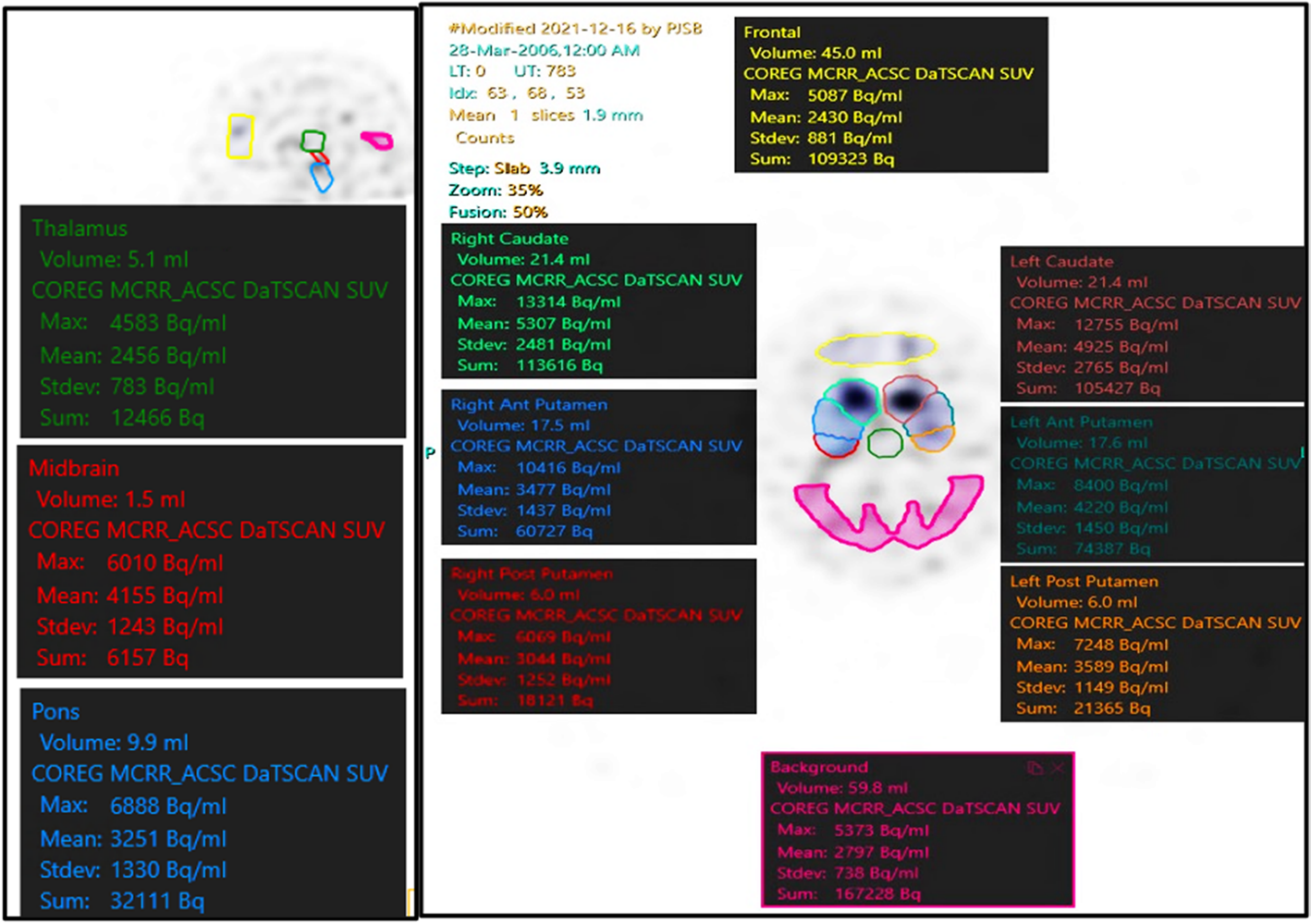
Sagittal (left) and axial (right) DaTSCAN with the modified Hermes EARL Ltd template used for VOI analysis. The colours represent different VOIs with analysis data presented in the same colour. Mean values had been used to calculate the absolute activity quantification of DaTSCAN binding, termed normalised concentration (NC)

VOIs were developed using an adapted version of the EARL Ltd (EANM Research 4 Life) template from Hermes which included the caudate and putamina (anterior and posterior). This adaption was constructed by expanding the original VOI map. Extra VOIs were added for this study and included the frontal region, thalamus, midbrain, pons and occipital background region using a T1-weighted MPRAGE MRI scan as anatomical descriptors. The concentration of radioactivity in each VOI was again corrected to the administered radiolabel activity to produce a normalised concentration (NC) (Fig. 1).

The formula used to calculate the NC for specific VOIs is stated below:

$${\text{Normalised }}\;{\text{Concentration}}\;{\text{ (NC)}} = \frac{{{\text{Mean }}\;{\text{Concentration}}_{{{\text{Relevant }}\;{\text{VOI}}}} [{\text{Bq/mL}}]}}{{{\text{Corrected}}\;{\text{ Administered}}\;{\text{ Activity}} [{\text{Bq}}]}}$$

### Statistical analysis

Statistical analysis was performed using IBM SPSS Statistics (version 27) for macOS Monterey (version 12.1). DaTSCAN availabilities were extracted from both SPECT (DaTQuant) and SPECT-CT images obtaining SBRs and NCs, respectively, for all VOIs. Pearson correlation was used to measure the linear relationship between SBRs/NCs and BMIs/weight within the whole cohort. This was repeated in the abnormal, borderline and SWEDD subgroups separately. The level of significance was set as *P* < 0.05.

## Results

**Fig. 2:**
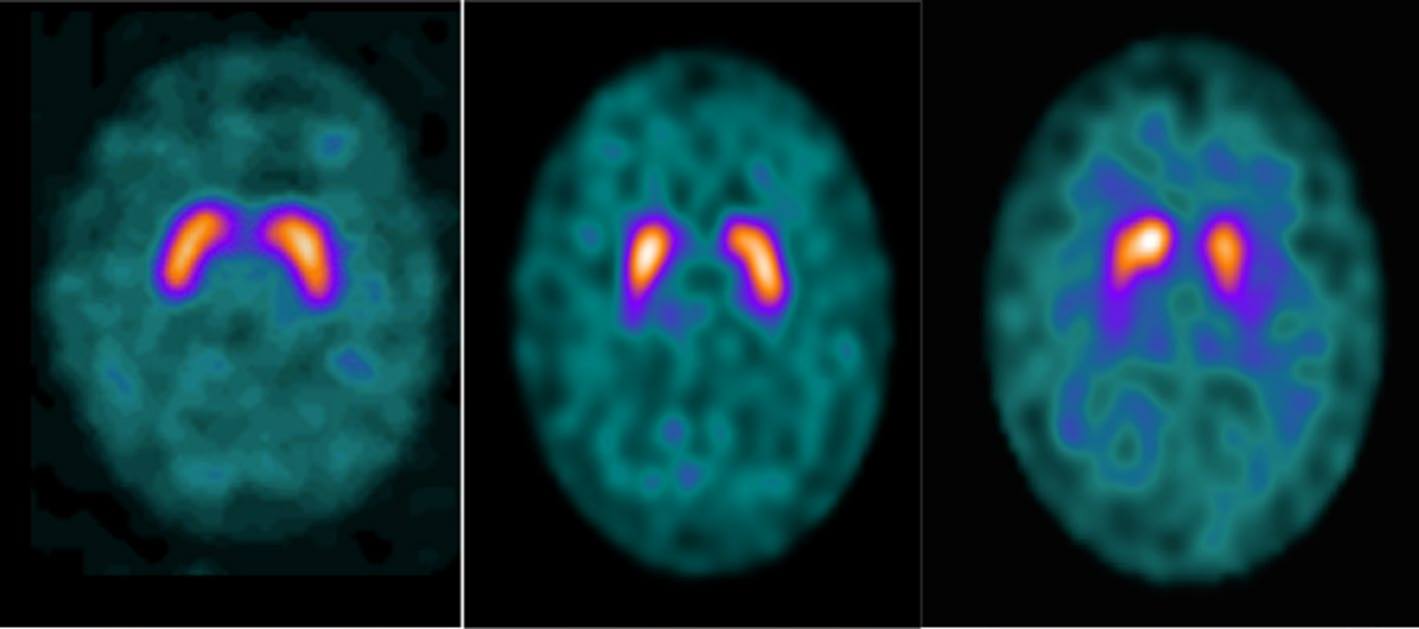
SWEDD (left), borderline (middle) and abnormal (right) [^123^I]Ioflupane (DaTSCAN) SPECT Scans. SWEDD demonstrates bilateral inverted comma shape representing the caudate nucleus (at the top) and putamen (at the bottom). Borderline image shows a suspicious “weak comma” on the right side of the brain scan but had normal SBR values. The abnormal scan shows an obvious left-sided “full stop”-shaped binding with diminished SBR in this area

Seventy-four patients with symptoms of parkinsonism, who had undertaken routine SPECT-CT DaTSCAN, were included in the analysis. The scans were also subdivided into either degenerative parkinsonism (abnormal = 49), borderline (*n* = 14) or scan without evidence of dopaminergic deficit (SWEDD = 11) according to both visual assessment and specific binding ratios (SBR) by nuclear medicine consultants (Table 1). The scans were categorised as borderline if images visually suggested reduced DaTSCAN binding within the striatum, with either borderline or lower range of normal DaTQUANT SBR values (Fig. 2).

### Semi-quantitative values (SBR) and absolute activity quantification (NC) with BMI

**Fig. 3:**
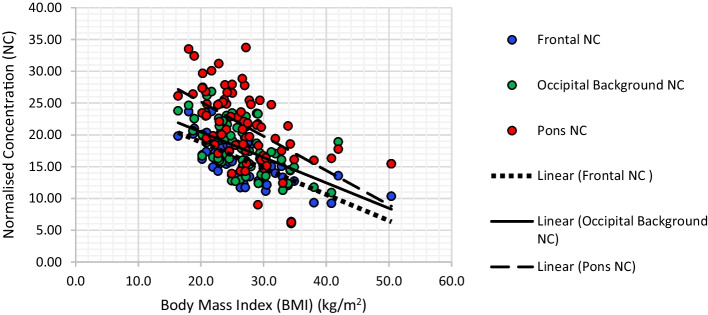
Scatter graph presenting the inverse correlation between increasing BMI and DaTSCAN availability in the frontal, midbrain and occipital background regions, measured by the absolute activity normalised concentration (NC). Blue = Frontal region; green = occipital background region; red = pons

DaTSCAN SBRs were not correlated with subject BMIs in any of the predetermined VOIs. In comparison, NC values correlated negatively in all VOIs (Table 2) with the strongest correlation seen in the frontal (*r* = − 0.649. *p* = 0.000), occipital background (*r* = − 0.555, *p* = 0.000) and pons (*r* = − 0.555, *p* = 0.000) (Fig. 3) regions.

**Table 2:** An inverse correlation between normalised concentrations and body mass indices

	NC within VOIs										
	F	RC	RAP	RPP	LC	LAP	LPP	OB	T	M	P
r	− 0.649**	− 0.370**	− 0.234*	− 0.354**	− 0.353**	− 0.283*	− 0.358**	− 0.555**	− 0.282*	− 0.395**	− 0.555*
Sig. (two-tailed)	0.000	0.001	0.045	0.002	0.002	0.015	0.002	0.000	0.015	0.000	0.000

Significance represented at either 0.05 level* or 0.01 level**

NC = Normalised concentration; VOI = volume of interest (F = frontal; RC = right caudate; RAP = right anterior putamen; RPP = right posterior putamen; LC = left caudate; LAP = left anterior putamen; LPP = left posterior putamen; OB = occipital background; T = thalamus; M = midbrain; P = pons); *r* = Pearson correlation; Sig. = significance. Sig of 0.000 represents significance of < 0.001

### Semi-quantitative values (SBR) and absolute activity quantification (NC) with weight

DaTSCAN SBRs displayed a weak positive correlation with subject weight in all striatal areas, within the whole cohort (Table 3). The striatal regions on the right showed more correlation than the left, with a Pearson correlation (r) value of + 0.354 (*p* = 0.002) within the right striatum. However, the relationship between weight and NC values demonstrated inverse correlations in all VOIs (Table 4). Like the linear relationship between NC and BMI, NC with the subject weights demonstrated the strongest correlations in the frontal (*r* = − 0.724, *p* = 0.000), occipital background (*r* = − 0.660, *p* = 0.000) and pons (*r* = − 0.598, *p* = 0.000) regions.

**Table 3:** A weak positive correlation between specific binding ratios (SBR) and subject weight (kg)

	SBR within VOIs									
	RS	LS	RP	LP	RAP	LAP	RPP	LPP	RC	LC
r	0.345**	0.287*	0.348**	0.276*	0.329*	0.265*	0.306**	0.256*	0.350**	0.287*
Sig. (two-tailed)	0.002	0.013	0.002	0.017	0.004	0.022	0.008	0.028	0.002	0.013

Significance represented at either 0.05 level* or 0.01 level**

SBR = Specific binding ratio; VOI = volume of interest (RS = right striatum; LS = left striatum; RP = right putamen; LP = left putamen; RAP = right anterior putamen; LAP = left anterior putamen; RPP = right posterior putamen; LPP = left posterior putamen; RC = right caudate; LC = left caudate); *r* = Pearson correlation; Sig. = significance. Sig. = Significance. Sig of 0.000 represents significance of < 0.001

**Table 4:** An inverse correlation between normalised concentrations and subject weight (kg)

	NC within VOIs										
	F	RC	RAP	RPP	LC	LAP	LPP	OB	T	M	P
r	− 0.724**	− 0.429**	− 0.268*	− 0.413**	− 0.432**	− 0.277*	− 0.317**	− 0.660**	− 0.360*	− 0.495**	− 0.598*
Sig. (two-tailed)	0.000	0.000	0.021	0.000	0.000	0.017	0.006	0.000	0.002	0.000	0.000

Significance represented at either 0.05 level* or 0.01 level**

NC = Normalised concentration; VOI = volume of interest (F = frontal; RC = right caudate; RAP = right anterior putamen; RPP = right posterior putamen; LC = left caudate; LAP = left anterior putamen; LPP = left posterior putamen; OB = occipital Background; T = Thalamus; M = Midbrain; P = Pons); *r* = Pearson correlation; Sig. = Significance. Sig. = Significance. Sig of 0.000 represents significance of < 0.001

### Subgroup results

**Fig. 4:**
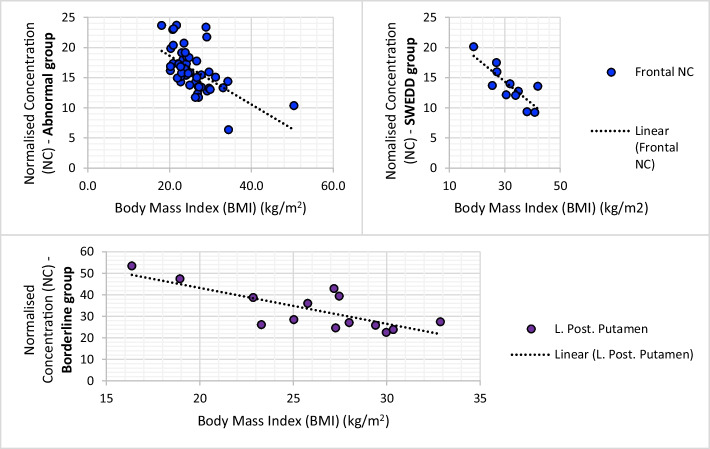
Scatter graph presenting the inverse correlation between increasing BMI and DaTSCAN availability in areas with the most significant correlations within respective subgroups (abnormal, borderline and SWEDD). Abnormal group (top left) and SWEDD group (top right) both display a negative relationship between BMI and frontal region. Borderline group scatter graph displays an inverse relationship between BMI and left posterior putamen. Blue = Frontal region; purple = left posterior putamen

SBR values obtained from the abnormal (*n* = 49), borderline (*n* = 14) and SWEDD (*n* = 11) subgroups did not correlate with either BMI or weight. However, significant inverse correlations were demonstrated between absolute activity quantification measures (NC) and BMI/weight (Table 5). In the abnormal group, NC of the frontal region was the most correlated with BMI (*r* = − 0.570, *p* = 0.000) while NC of the occipital background region was most correlated with the weight (*r* = − 0.689, *p* = 0.000). Within the borderline group, the NC within the left posterior putamen was most associated with BMI (*r* = − 0.765, *p* = 0.001) with the frontal region also demonstrating a strong inverse correlation (*r* = − 0.656, p < 0.05). The frontal region NC was also most correlated with weight (*r* = − 0.622. *p* = 0.018) in the borderline group. The SWEDD group showed the strongest association in the frontal region when correlating NC with both BMI (*r* = − 0.813, *p* = 0.002) and weight (*r* = − 0.941 *p* = 0.000) (Fig. 4).

**Table 5:** An inverse correlation between normalised concentrations and subject BMI/weight (kg)

	NC within VOIs										
	F	RC	RAP	RPP	LC	LAP	LPP	OB	T	M	P
*BMI (r)*											
A	− 0.570**	− 0.422**	− 0.377**	− 0.463**	− 0.439**	− 0.540**	− 0.415**	− 0.516**	− 0.286*	− 0.425**	− 0.552**
B	− 0.656*	− 0.711**	− 0.545*	− 0.581*	− 0.375	− 0.572*	− 0.765**	− 0.682**	− 0.096	− 0.124	− 0.558*
S	− 0.813**	− 0.533	− 0.558	− 0.195	− 0.440	− 0.595	− 0.696*	− 0.502	− 0.133	− 0.524	− 0.651*
*Weight (r)*											
A	− 0.687**	− 0.525**	− 0.474**	− 0.575**	− 0.543**	− 0.591**	− 0.461**	− 0.689**	− 0.364*	− 0.564**	− 0.640**
B	− 0.622*	− 0.615*	− 0.563*	− 0.542*	− 0.395	− 0.494	− 0.530	− 0.448	− 0.056	− 0.124	− 0.452
S	− 0.941**	− 0.740**	− 0.730*	− 0.296	− 0.607*	− 0.746**	− 0.747**	− 0.738**	− 0.367	− 0.764**	− 0.793**

Significance represented at either 0.05 level* or 0.01 level**

NC = Normalised concentration; VOI = volume of interest (F = frontal; RC = right caudate; RAP = right anterior putamen; RPP = right posterior putamen; LC = left caudate; LAP = left anterior putamen; LPP = left posterior putamen; OB = occipital background; T = thalamus; M = midbrain; P = pons); *r* = Pearson correlation; A = abnormal scan group; B = borderline scan group; S = scan without evidence of dopaminergic deficit group

## Discussion

To the best of our knowledge, this is the only study that investigates the effect of BMI on [^123^I]Ioflupane (DaT/SERT) availabilities in both striatal and extrastriatal regions using an absolute activity quantification method. A previous Brighton and Sussex Medical School independent research project, where the SPECT-CT methodology was developed, reported a strong inverse correlation between DaTSCAN binding and weight (Missir et al. 2023). However, the project was conducted with a small sample size (*n* = 26) and BMI had not been investigated. In this current study, NC values consistently show an inverse correlation with both BMI and weight, and this is particularly seen in the extrastriatal regions.

The imbalance between energy intake and energy expenditure can be explained by the lack of control of the homeostatic pathway, coordinated by serotonin transmission (Lam et al. 2010) and alteration of DA signalling within the reward circuitry (Blum et al. 2014). DA is a neurotransmitter that normally relays reward signals in response to food stimuli (Morton et al. 2014) and is mainly situated within the striatum (Stice et al. 2013). Habituation is induced with repeated exposure to food reward (Schultz 2010) and leads to the overconsumption of palatable foods (Stice et al. 2008). This response eventually overrides the regulation of satiety, resulting in the development and maintenance of obesity (Volkow et al. 2011).

DaTs regulate the levels of cerebral DA by controlling its reuptake from the synaptic cleft back to the presynaptic neuron (Zahniser and Doolen 2001), and hence, several previous studies have focused on the relationship between BMI and DaT (Nam et al. 2018; Versteeg et al. 2017; Chun Hsieh et al. 2010; Thomsen et al. 2013; Epstein et al. 2002). Only one study using (^99m^Tc)-TRODAT-1 SPECT reported a significant but moderate inverse correlation of − 0.44 (Chun Hsieh et al. 2010). This is consistent with our findings of a weak/moderate correlation between striatal DaT and BMI. Another study on healthy volunteers by Chen et al. showed an inverse correlation between the increase in weight and DaT levels in the striatum (Chen et al. 2008), which was supported by Wu et al. in a post-mortem human study (Wu et al. 2017), again providing support to the association of DaT binding and regulation of weight. In contrast, another in vivo DaTSCAN SPECT study on healthy subjects demonstrated no correlation between the two variables (Giessen et al. 2013), which may be due to the use of the relative binding ratios (semi-quantitative analysis) to measure DaT availability instead of absolute activity values, like in this study.

Second-generation antipsychotic drugs which modulate the neurotransmitter are also known to increase the risk of obesity and other metabolic syndromes (Newcomer 2005). However, the mechanism is again unclear; this study does suggest DaT dysregulation may be the underlying cause of the effects seen in antipsychotic medication, thus resulting in weight gain. Extrapolating this theory, these data may also suggest that long periods of adaptations in DaT function may be the underlying cause in DA dysregulation making an individual more vulnerable to overeating to satisfy their craving for pleasurable food. However, this research has reported a weak correlation within the striatum at the presynaptic level and thus may indicate a stronger involvement of other factors including extrastriatal regulation of 5-HT or even other neurotransmitters (Palmiter 2007) or DA receptors at the post-synaptic level (Eisenstein et al. 2013).

Interestingly, in all the subdivided groups (abnormal, borderline and SWEDD), the NC values within left putamen displayed a significant inverse correlation with BMI. The correlation was strongest within the borderline group, potentially suggesting this region is involved in the pathogenesis of their parkinsonism. However, this finding was also seen in the SWEDD group which has been used to represent the “normal” population in this study. Therefore, this finding may also suggest shared DA and 5-HT networks within the basal ganglia as suggested by previous molecular studies (Lin et al. 2011; Aggarwal and Mortensen 2017).

In addition to the negative correlation seen with striatal DaT availability and BMI, this study shows a greater association between extrastriatal NC and obesity, likely due to SERT dysfunction. 5-HT is known to be localised in the brainstem and networks project across the entire brain, with a high density of serotoninergic axons situated in the frontal lobe (Celada et al. 2013). There are non-human studies that suggest the presence of DA fibres within the prefrontal cortex (Chaua et al. 2018); however, 5-HT is known to be a major modulator for high executive tasks within the frontal lobe as suggested by the prominent expression of 5-HT receptors and abundant innervation by 5-HT neurons (Puig and Gulledge 2011). Assuming most of the signal is due to 5-HT, there is a continuous interaction between synaptic 5-HT and expression of SERT, and hence, the decrease in 5-HT causes a downregulation of the transporters (Rothman et al. 2003). Supporting this, the current study indicates the strongest association between the frontal regions and increasing BMI, suggesting the possible involvement of this area in obesity.

An inverse correlation had also been reported between SERT and BMI using the selective cerebral [^11^C]-3-amino-4-(2-dimethylaminomethyl-phenylsulfanyl)-benzonitrile (11C-DASB) radiotracer within the striatum, midbrain and global neocortex (Erritzoe et al. 2010), again supporting our findings. However, the reported relationship between SERT and BMI has also been highly inconsistent with one study reporting a higher SERT binding correlation with high BMIs when comparing transporter availabilities between monozygotic twins (Koskela et al. 2008). This positive correlation was also reported within the thalamus of a voxel-based analysis using ^123^I-FP-CIT (Hesse et al. 2014), which again is inconsistent with the findings in this research which has demonstrated the least negative correlation within the thalamus, potentially explained by the complexities of the cohort used in this study. All the patients had parkinsonism symptoms and previous research has suggested both Parkinson’s disease (PD) and dementia with Lewy bodies (DLB) have lower SERT binding in the hypothalamus (Joling et al. 2019) and thalamus region (Pagano et al. 2017), suggesting our results demonstrate the expected neurodegeneration pattern seen in such diseases.

Additionally, no correlation between BMI and SERT had been deduced in studies conducted by both Hesse et al. (2014) and Versteeg et al. (2017), who investigated the relationship between using relative semi-quantitative analysis. Another comparative DaTSCAN study by Nam S et al. reported a positive association within the pons but also both a negative and positive correlation in the midbrain between SBRs and BMI in obese and non-obese subjects, respectively (Nam et al. 2018), suggesting a more complex relationship may exist. The pons region in our study also suggested an association; however, this was an inverse relationship. Despite the contradictory results, these studies may indicate an important involvement of the pons in regulating weight.

The inconsistency and controversy in the results of these previous studies may be due to the use of relative semi-quantitative methods used to represent target binding. This discrepancy in results is also demonstrated in this research when using relative quantification, with whole-cohort SBRs reporting a weak positive correlation with weight, but no correlation with BMI. The more accurate NC values (absolute activity quantification) showed consistent negative correlations with both weight and BMI, within the entire cohort and in the subdivided groups. The reliability of these results is highly reliant on the accuracy of the quantification method (Schepper et al. 2021). As absolute activity quantification is more reliable and precise, it represents a more accurate correlation to transporter availabilities. A previous phantom study had tested the accuracy of absolute activity quantification in (^99m^Tc)-TRODAT-1 SPECT-CT and reported comparable quantification to positron emission tomography–CT (PET-CT) systems, which are considered the current gold standard modality for absolute quantification (Gnesin et al. 2016). A similar validation study is therefore suggested for DaTSCAN SPECT-CT to establish its value in clinical practice.

The inverse association established between BMI and [^123^I]Ioflupane uptake, which infers extrastriatal SERT availability in this study, could be potentially explained by either genetic and/or environmental factors that directly or indirectly cause a change in cerebral SERT levels. Previous animal research studying SERT knockout mice has shown the development of obesity at the age of approximately 3 months (Murphy and Lesch 2008). Additionally, polymorphism of the short allele of SLC6A4 which encodes the SERT has been associated with obesity (Sookoian et al. 2007), and molecular imaging has supported the role of genetics in 5-HT regulation by reporting higher SERT binding in homozygotic carriers of the two long alleles of the SERT within the midbrain (Reimold et al. 2007). Environmental factors are also known to manipulate the regulation of cerebral 5-HT; for example, restriction of protein during perinatal stages of development reduces the inhibitory action of 5-HT on food stimuli and contributes to developing obesity in adulthood (LopesDeSouza et al. 2008). While this suggests the involvement of 5-HT in the regulation of obesity, the mechanism remains unclear and is potentially a result of numerous complex environmental and genetic interactions which may predispose the likelihood of obesity.

Lastly, this study identified no relationship between SBR and BMI. This can potentially be explained by the effects of obesity on the measured values. As BMI increases, the results using absolute activity quantification suggest NC within the occipital background region decreases. With low DaTSCAN uptake in the occipital background region, the calculated SBRs (Sect. 4.4.2.1) will increase and adversely affect the interpretation of the scan. Over 70% of the borderline group had a BMI of more than 25 kg/m^2^, classifying them as either overweight or obese. Thus, the reduced DaTSCAN binding in the occipital background could explain the high proportion of borderline scans in this study. The presence of low, albeit measurable, levels of SERT in the occipital cortex (Laruelle et al. 1988; Bäckström et al. 1989) and in post-mortem (Kish et al. 2005) brain has previously been demonstrated. This is supporting the use of absolute activity values rather than relative ratios, as changes in occipital cortical SERT could interfere with binding ratios and may lead to a misinterpretation of results if the occipital cortex is taken as “SERT-free reference area/background”. Furthermore, previous studies in healthy human subjects have also shown that [^123^I]ioflupane achieves a state of equilibrium binding in the brain by 2–4 h post-injection. Since imaging occurs under equilibrium conditions, factors such as cerebral blood flow should not account for the increased NC levels in the occipital region.

The borderline SPECT images showed a typical pattern of either a “weak comma” (Fig. 2) or balanced loss (bilateral weakened DaTSCAN uptake and increased background activity) within the striatum, with a borderline or within the lower range of normal DaTQUANT SBR values, resulting in an unclear diagnosis. Due to the high precision of absolute activity quantification in contrast to relative quantitative analysis (SBRs), NC values may aid the diagnosis process of such highly complex patients. Thus, further research in this population group should be conducted to investigate the use of NC values in the diagnosis of atypical parkinsonism, particularly in patients where clinical diagnosis is uncertain.

### Limitations

This study has several limitations. First, the sample included patients presenting with parkinsonism symptoms. The majority included in this study are from abnormal and borderline groups and already display signs of dopaminergic loss within the striatum, so a decrease in DaTSCAN availability may well be related to DaT dysfunction due to neurodegenerative parkinsonism. Despite the inverse correlation reported in the SWEDD group, which included subjects with non-degenerative parkinsonism, these subjects also presented with some symptoms of parkinsonism, and thus, findings may not entirely be extrapolated in the general population.

Secondly, the accuracy of the VOI map used in this study has not been tested and may not account for individual variations in anatomy or may overlap brain regions that are close in proximity. Future studies may consider testing the VOI map used in this study by overlaying the template over numerous brain imaging with known anatomical landmarks and high resolution to assess its accuracy. Also, the frontal region and occipital background used in the VOI map represent a section of the frontal and occipital lobe, respectively, and the whole regions are not included in this study.

Thirdly, this study only investigated the associations between neuroimaging findings and BMI and weight; hence, a causal relationship could not be established. That is whether the dysregulation of DaT/SERT precedes or is a consequence of obesity. It is possible for an external factor to contribute to our findings, supported by a recent PET study reporting a negative correlation between SERT availability and BMI but only following glucose loading (Pak et al. 2022).

Fourthly, although this research has used both weight and BMI to represent obesity, these may not be related to levels of body fat as it does not directly measure adipose tissue within an individual (Pak et al. 2016). Both muscle and bone are denser than body fat; thus, BMI can be overestimated in athletic individuals with high muscle mass or underestimated in the elderly who have low bone density and low muscle mass. Also, this study has assumed that all binding in the striatal and extrastriatal regions represents DaT and SERT availability, respectively, and is not influenced by blood flow to the brain due to the high affinity of the ioflupane for DaT/SERT.

Additionally, previous studies have demonstrated an inverse correlation between SBR and age (Karrer et al. 2017). However, the dataset used in this study has a similar mean age when considering the different BMI categories. The mean age of individuals with a BMI of under 25 kg/m^2^ (healthy/underweight) was 73.2 (*n* = 32; range 32–89). The mean age of participants with a BMI of over 25 kg/m^2^ (overweight/obese) was 69.0 (*n* = 42; range 40–90). This shows that in our study patients with higher BMI had a similar or even lower mean age than the comparative group, therefore mitigating the potential effect the age may have had on the correlation performed in this study.

Finally, various concurrent illnesses (Haase et al. 2021) and medications (Serretti et al. 2010; Ahmed et al. 2022) have been associated with changes in BMI and some of these factors have not been considered in this study.

Future studies should eliminate above potential confounding variables to establish a more reliable association between BMI and DaTSCAN availability.

## Conclusion

DaTSCAN SBR values are relative and unable to absolutely quantify DaT/SERT availability within the brain. With the use of absolute NC measure, a strong inverse correlation with BMI has been demonstrated, strongest in the extrastriatal regions, particularly in the frontal region and least in the thalamus. Due to the predominately non-overlapping distribution of DaT and SERT, this study suggests greater involvement of SERT in obesity with possible interplay with DA transmission. It has not yet been established whether this decrease has a direct role in the regulation of eating behaviour or if this is due to a secondary cause in the respective systems. Thus, larger prospective studies with healthy subjects and pharmacological intervention are needed to investigate the full value of absolute activity quantification and the relationship between 5-HT neurotransmission, dopamine dysfunction and obesity. Further nuclear medicine imaging research should also investigate the use of NC values in diagnosing complex cases of parkinsonism with atypical features on DaTSCAN which could potentially provide an accurate and reliable diagnosis.

## Data Availability

The datasets used and/or analysed during the current study are available from the corresponding author on reasonable request.
